# Evaluating Preconception Health and Behaviour Change in Australian Women Planning a Pregnancy: The OptimalMe Program, a Digital Healthy Lifestyle Intervention with Remotely Delivered Coaching

**DOI:** 10.3390/nu16010155

**Published:** 2024-01-03

**Authors:** Bonnie R. Brammall, Rhonda M. Garad, Helena J. Teede, Cheryce L. Harrison

**Affiliations:** Monash Centre for Health Research and Implementation, Monash University and Monash Health, Melbourne, VIC 3168, Australia; bonnie.brammall@monash.edu (B.R.B.); rhonda.garad@monash.edu (R.M.G.); helena.teede@monash.edu (H.J.T.)

**Keywords:** preconception, pregnancy, women’s health, behaviour change, lifestyle, nutrition, physical activity, digital health, health coaching

## Abstract

OptimalMe is a digital healthy lifestyle intervention for women planning a pregnancy, with remotely delivered coaching. This follow-up study of Australian women, stratified by coaching delivery mode (phone vs. videoconferencing), assessed alignment to preconception care guidelines and self-reported behaviour change. Overall, 298 women enrolled with a mean (SD) age of 31.8 (4.3) years and mean BMI of 25.7 (6.1) kg/m^2^. Suboptimal preconception behaviours were reported at baseline, including alcohol consumption (57.2%), infrequent weighing (37.2%) and incomplete cervical cancer screening (15.8%) and prenatal supplementation (38.5). At follow-up (4.5 months) (*n* = 217), a statistically significant shift towards desired behaviours was reported for alcohol consumption (z = −2.6045, *p* = 0.00932), preconception supplementation (z = −2.7288, *p* = 0.00634) and frequent weight monitoring (z = −5.2911, *p* < 0.00001). An insignificant shift towards adherence to cervical cancer screening (z = −1.8679, *p* = 0.06148) was observed, with a positive trend towards adherence. Results indicate that women who are actively planning a pregnancy require support to optimise health and lifestyle in preparation for pregnancy and general health and lifestyle improvement. Women demonstrated improvement in lifestyle behaviours and self-monitoring, indicating the uptake of low-intensity, non-prescriptive information provision. Supporting the provision of knowledge-enhancing tools and general healthy lifestyle information combines with skilled health coaching as an effective method for behaviour change and self-management. OptimalMe also shows significant improvements in rates of healthcare engagement, which suggests coaching-based digital health interventions may decrease women’s barriers for preconception care and improve engagement in clinical settings.

## 1. Introduction

The health of women prior to pregnancy significantly influences fertility, pregnancy and intergenerational health outcomes [1,2,3,4,5]. The increasing prevalence of overweight, obesity and suboptimal weight-related behaviours in reproductive aged women are major public health concerns [6,7]. Excess weight prior to conception is associated with increased risk of adverse maternal and neonatal outcomes, including gestational diabetes mellitus, caesarean section, macrosomia and delivery of a large-for-gestational-age infant. These risks are also independently exacerbated by excessive weight gain during pregnancy [8]. Due to the detrimental impacts these risk factors have on reproductive health [1,9,10] and maternal and child health outcomes [1,9,10], finding an effective approach to deliver interventions that promote behaviour change in reproductive aged women is imperative.

Whilst pregnancy is established as an opportune window to optimise maternal health and lifestyle behaviours [11], there is increasing recognition that intervening during pregnancy may be too late to positively influence the many critical epigenetic processes that occur before conception or in the very early stages of pregnancy [1]. Consequently, in women with an intention to conceive, improving health and wellbeing in the time before pregnancy is favourable. During this time, women may have heightened motivation and increased readiness to optimise health behaviours that benefit conception, pregnancy health outcomes and the health of their baby [11]. The reduction or cessation of high-risk behaviours such as tobacco smoking and alcohol consumption have been successful in women who wish to fall pregnant or are pregnant [12,13], supporting this period as a significant window of opportunity for behaviour change. Harnessing this motivation prior to an intended pregnancy allows women adequate time to optimise behaviour, improve health and implement self-monitoring strategies.

Preconception care (PCC) comprises counselling and interventions that aim to detect and change biomedical, behavioural and social risks to optimise the health of women prior to pregnancy to improve maternal and neonatal health outcomes [14]. The preconception period and PCC encompass three domains [14]: firstly, the biological perspective, which includes the days to weeks before embryo development [14]; secondly, the individual perspective, which comes after a decision to conceive, typically weeks to months before pregnancy occurs [14]; finally, the public health perspective, which encompasses longer periods of months or years to address preconception risk factors, such as diet and obesity [14]. Despite recognising the importance of enhancing health behaviours among women of reproductive age, there remains limited progress at a population level. One of the primary challenges involves effectively engaging women in PCC, especially those not regularly involved with healthcare services. Currently, the focus of most interventions has centred on higher-risk groups, including individuals with medical conditions or infertility [15,16,17,18,19,20], inadvertently overlooking a significant portion of women. Understanding how and where to reach women from a general population for PCC has been a widely considered topic. Notably, a recent study revealed that 80% of women favoured responding to inquiries about future pregnancies online and receiving online tailored advice accordingly [21]. This highlights the potential of leveraging the internet to provide accessible information, nurturing proactive engagement among otherwise healthy women in prioritising their reproductive health. Additionally, from a public health perspective, digital interventions offer a cost-effective and scalable means to disseminate information, foster healthy behaviours and gather data for informed decision-making, aiming to improve overall population health outcomes during preconception.

OptimalMe is a co-designed, coaching-based, digital health intervention that aims to meet the unique preconception needs of women who intend to conceive. OptimalMe aims to target an otherwise healthy population of women to initiate PCC holistically, addressing preventative clinical care and relevant lifestyle behaviours during preconception, pregnancy and postpartum. Here, we aim to explore the impact of OptimalMe on secondary outcome measures, encompassing self-reported behaviour change during preconception, and compare the impact of different delivery modes (phone and videoconferencing) on behaviour change outcomes.

## 2. Methods

### 2.1. Study Design

OptimalMe is a type III hybrid effectiveness–implementation study [22]. The intervention is a parallel, two-arm randomised trial at the level of the individual. Women receive the same intervention yet are randomised into two groups for remotely delivered health coaching (phone and videoconferencing). Detailed study design and methodologies have been previously published [23].

### 2.2. Population, Eligibility Criteria and Recruitment

In brief, the target population for OptimalMe were female members of one of Australia’s largest private health insurance providers, Medibank Private, who joined or upgraded with pregnancy and birth coverage within three months prior to recruitment, who were not pregnant but wished to conceive within 12 months, aged 18–44 years, that read and spoke English and had access to a digital device (i.e., mobile phone and/or desktop computer) with internet access. A co-designed process with Medibank Private was developed to facilitate Australia-wide recruitment using an opt-in design with women randomly allocated to one of two coaching delivery modes. Complete details regarding design, eligibility criteria, sample size, randomisation and recruitment are published elsewhere [23].

### 2.3. Intervention Overview

OptimalMe is underpinned by our previous healthy lifestyle program, HeLP-her [24,25,26,27,28], a low-intensity behaviour change program grounded in social cognitive theory [29] which effectively optimises weight and lifestyle related behaviours. The intervention is designed to be non-prescriptive with simple messages on healthy eating and physical activity aligned with national guidelines [30,31,32]. Preconception information and outcome measures were informed by the Royal Australian College of General Practitioners (RACGP) guideline for ‘preventive activities prior to pregnancy’ [14]. Behaviour change is iteratively practiced through identifying health priorities and needs, goal setting, action planning, problem solving and self-monitoring, supported by a health coach.

OptimalMe is a digital program, incorporating preconception health and lifestyle education, aligned with national dietary, physical activity and perinatal guidelines [14,30,31,32,33,34]. Preconception health information is complemented by digital resources to promote self-monitoring (e.g., preconception health checklists, body mass index [BMI] calculator) and a goal setting tool to set and review action plans. The digital program is supported by two personalised coaching sessions of approximately 20 min at two to four and ten to twelve weeks post commencement. Individual coaching sessions focused on goal setting, problem solving, and self-management, and reinforced program messages and objectives. Coaching sessions were arranged by email and a reminder email was sent prior to the appointment. If participants did not attend their session after two phone calls or after waiting 10 min in the videoconferencing platform, they were contacted via email to reschedule. Participants were rescheduled up to two times, and then the coach sent a session overview via email if they did not attend or did not respond.

### 2.4. Outcome Measures

Quantitative questionnaires were completed autonomously by participants at baseline on the OptimalMe digital platform and after completion of the preconception intervention (evaluation) via an external digital survey platform, with a link and reminders sent via text and email. Questionnaires included demographic information (i.e., age, country of birth (COB), ethnicity, marital status, working status, household income, etc.). The baseline and evaluation questionnaires aligned with Australian PCC recommendations [14]. These included reproductive history (i.e., parity and previous pregnancy outcomes); genetic screening; general physical assessment (i.e., weight, height, chronic disease history and cervical screening history); screening for immunisation status; folate/folic acid and iodine supplementation; self-weighing frequency, macronutrient food group intake; physical activity and sedentary behaviours; and substance use (i.e., tobacco, alcohol and recreational drugs) [14,35].

Self-reported weight and height were used to calculate BMI (weight/height (m^2^)), which was classified according to the World Health Organization (WHO) definitions: underweight (≤18.50 kg/m^2^); normal-weight (18.50–24.99 kg/m^2^); overweight (25.00–29.99 kg/m^2^); and obese (≥30.00 kg/m^2^) [36]. Self-weighing behaviours were classified as frequent (i.e., daily, weekly or monthly weighing) or non-frequent (i.e., occasional or never weighing).

Current and/or recent behaviour relating to alcohol consumption, recreational drug use and tobacco smoking was collected. Tobacco use was recorded by asking ‘do you currently smoke’ (yes/no/no, I stopped for pregnancy), and alcohol consumption at baseline was recorded by asking ‘do you currently drink alcohol’ (yes/no/no, I stopped for pregnancy). Then, at the preconception evaluation, women were asked ‘since starting OptimalMe have you: smoked, drunk (any) alcohol, consumed four or more drinks in a single occasion’ (yes/no).

### 2.5. Analyses

Data analysis was performed using IBM SPSS Statistics version 25 (Armonk, NY, USA). Frequencies and percentages are presented for categorical variables. Descriptive statistics were tested for skewness by using the Shapiro–Wilk test and are presented as mean and standard deviation (SD) for normally distributed variables. The Kruskal–Wallis test, Mann–Whitney U test or the chi-squared test (χ^2^ tests) were used to compare the characteristics of participants stratified by health coaching delivery groups. All *p*-values presented are two-tailed; *p* < 0.05 was considered statistically significant. Where a significant *p*-value was identified in a multiple comparison, the Bonferroni correction was used to examine whether the significance remained after adjusting for multiple groups [37]. Response rates varied for each question, and therefore, numbers differ throughout the results. Little’s Missing Completely at Random (MCAR) [38] analysis for evaluation data was conducted based on key demographics (age, BMI, COB, ethnicity, education, income, work status and marital status).

Individual participant data were examined to determine whether women who reported undesired behaviour at baseline changed to desired behaviour after the intervention. Subsequently, behaviour change analysis included a test of overall sample proportions (z-score calculation). This analysis examines the proportion of whole sample behaviours at baseline and evaluation.

### 2.6. Ethics

The Monash Health Human Research and Ethics Committee approved the study (date: 4 September 2019, reference: RES-19-0000291A), which has been registered on the Australian and New Zealand Clinical Trial Registry (ACTRN12620001053910). Participants provided written, informed consent to take part.

## 3. Results

### Participants

Overall, 527 women expressed interest to participate. Of these, 33 did not meet the inclusion criteria and a further 196 failed to engage after expressing interest, leaving 298 overall who were enrolled in OptimalMe and randomised to coaching delivery groups (phone *n* = 153 and videoconferencing *n* = 145). The mean age of the recruited cohort was 31.8 (4.3) years, and the majority were born in Australia (70.8%) and of Oceanian or European ethnicity (39.9% and 26.8%, respectively). Most women were married or in a de facto relationship (92.6%), had no children (86.2%), were highly educated (80.5% held a bachelor's degree or above) and in fulltime work (77.2%). No significant baseline differences in demographic characteristics were found between the health coaching groups (Table 1).

Compared with key demographic characteristics from the Australia Bureau of Statistics [39], 38.6% of our cohort reported a higher household income than the population median (AUD 2329/week) [40]. The frequency of those reporting unemployment (6.7%) was comparable to Australian females aged 15 years and over (6.7% unemployed) [40]. A similar portion of women in this study were born overseas, compared to the overall Australian population (29.2% vs. 29.1%) [40].

## 4. Baseline Preconception Health and Behaviour

Overall, mean BMI at baseline was 25.7 (6.1) kg/m^2^, with 54.7% (*n* = 172) of women classified as having a healthy BMI and 62.8% reporting regular self-monitoring of weight. Approximately 50% of women reported no chronic conditions or relevant medical conditions at baseline. Anxiety (22.1%), asthma (13.4%), depression (12.8%) and polycystic ovary syndrome (PCOS) (12.4%) were the most frequently reported conditions. Approximately 10% of women reported a previous pregnancy loss, and 15.8% of women did not have up-to-date screening for cervical cancer prevention in accordance with Australia’s National Cervical Screening Program (NCSP) [41]. No significant differences in general and reproductive health outcomes were found between the two groups (Table 2).

At baseline, 57.2% of women reported recently consuming alcohol, while 15.8% had stopped consumption to prepare for pregnancy. The incidence of smoking and recreational drug use was low (1.7% and 0.3%, respectively). Almost 40% were yet to initiate preconception supplementation, while 67.3% were not using contraception. No significant differences were observed between the two groups (Table 2).

### Post-Intervention Preconception Health and Lifestyle Behaviour Change

The OptimalMe evaluation was completed by 217 women, 72.8% of the study population, an average of 4.5 months after commencing the intervention. Using demographic information, evaluation data (27%) were found to be missing completely at random (*p* = 0.112); therefore, the imputation of missing data was negated.

Following the intervention, the overall frequency of women engaging in frequent self-monitoring of their weight had increased to 84.1%. Almost three quarters (72.8%) of women reported that they had visited a general practitioner (GP) in preparation for pregnancy, 45.6% had drunk alcohol (any) and 12.4% had excessively drunk (four or more standard drinks in a single occasion). Eighty-seven percent (86.6%) of women indicated that they had improved their diet (increased fruit or vegetable intake or decreased discretionary food intake), physical activity (increased physical activity or decreased sedentary behaviour) and/or another personally defined goal area (e.g., including but not limited to improving sleep habits, reducing stress, increasing water consumption or reducing alcohol consumption). Half of women believed that completing the intervention had improved their knowledge relating to healthy food choices (50.0%), unhealthy food choices (40.7%) and ways to be physically active (48.1%). No significant differences were found between groups (Table 3).

Of those who provided an evaluation that were infrequently weighing at baseline (*n* = 81), 64.2% (*n* = 52) had adopted frequent weight monitoring, and 57.1% (*n* = 44) of the respondents not taking a preconception supplement (*n* = 77) had initiated supplementation. Those with incomplete cervical cancer screening (*n* = 35) demonstrated a 42.9% (*n* = 15) positive change in their screening status since participating in OptimalMe. Finally, over a third (35.8%, *n* = 42) of those who drank alcohol at baseline (*n* = 117) had not consumed any alcohol since the intervention, and 78.6% (*n* = 92) reported that they had not drank excessive amounts (four drinks in a single occasion). The proportion of participants reporting the desired behaviours was compared at baseline and evaluation via a test of proportions. At follow-up, a statistically significant shift towards desired behaviours was reported for alcohol consumption (z = −2.6045, *p* = 0.00932), preconception supplementation (z = −2.7288, *p* = 0.00634) and frequent weight monitoring (z = −5.2911, *p* < 0.00001). An insignificant shift towards adherence to cervical cancer screening (z = −1.8679, *p* = 0.06148) was observed, with a positive trend towards adherence.

## 5. Discussion

The OptimalMe study is the first to examine the impact of a digital health intervention with remotely delivered coaching to a general, otherwise healthy female population with the intention to conceive. Our findings demonstrate divergence from PCC objectives [14], as previously shown in Australian women planning a pregnancy [42]. Our evaluation supports the provision of PCC education and remotely delivered health coaching as an effective strategy for optimising women’s health, with improved adherence to preventative preconception health actions and lifestyle behaviours, and a considerable number of primary care consultations to prepare for pregnancy.

The OptimalMe cohort consisted of women with private health insurance who were otherwise healthy, with low incidence of chronic diseases or relevant medical history. Despite this, many preconception health behaviours were suboptimal. Women with private health insurance signal an intention to conceive by upgrading to or joining a policy that includes pregnancy care. These women are subject to a 12-month waiting period before a pregnancy-related insurance claim can be made. This provides unique insight into the individual perspective of preconception, when a decision to conceive is made, and provides a window of opportunity for intervention in the months before pregnancy. Exploring the impact of OptimalMe in this general population allows us to understand how those who have decided to conceive are preparing for pregnancy and determine if there are opportunities for health promotion. Whilst this group are generally of a high socioeconomic status (SES) and education, our cohort aligns with a large portion of the female population, as ~50% of Australian women of reproductive age have private health insurance and 25% birth in private hospitals [43]. Baseline reporting emphasised a range of opportunities for change such as alcohol consumption, excess weight, infrequent weighing, incomplete supplementation and immunisations and not engaging with PCC before ceasing contraception. The majority of women presented with opportunities for lifestyle or clinical improvement. This suggests that higher SES and education are not protective or predictive indicators of optimal preconception health. Our baseline results align with previous research showing that suboptimal PCC is common in the general population and strengthens the need for interventions to improve awareness of PCC and preventative health prior to pregnancy to all women of reproductive age, irrespective of health status and demographic factors.

Previous research has identified barriers for engagement with clinical care to prepare for pregnancy. These include a lack of health care engagement due to ambivalence in planning for pregnancy, uncertainty of timeline to conception, perceived absence of risks and lack of awareness of PCC [44]. Preconception and digital health interventions targeting women with diabetes indicate that interventions can significantly improve attitudes toward seeking PCC and reduce relevant barriers [20]. Additionally, the provision of a web-based preconception education module prior to attending a scheduled women’s health appointment has been shown to significantly increase the proportion of women discussing reproductive health [45], thereby promoting preconception health awareness. Similarly, OptimalMe encouraged health care engagement by providing women with a checklist for preconception actions to address and promoted partnership with their primary health care provider. Action items included discussing fertility optimisation and genetic screening; reviewing supplements, medications and medical conditions; and checking cervical screening requirements, immunisation status and contraception. A cross-sectional study in a similar population reported that only 40% of women planning a pregnancy had sought health or medical advice for pregnancy preparation [42]. Following the OptimalMe intervention, 73% of women had visited a GP to prepare for pregnancy. Compliance with cervical screening improved by 43% in women whose screening was not in accordance with the NCSP at baseline. OptimalMe shows significant improvement in rates of healthcare engagement, which suggests coaching-based digital health interventions may decrease women’s barriers for PCC and improve engagement in clinical settings.

The OptimalMe preconception intervention improved lifestyle-related knowledge and behaviours and decreased high-risk behaviours, with significant proportional shifts towards desired behaviours. A large proportion adopted frequent weighing behaviour from infrequent weighing at baseline. Given the benefit of self-weighing for weight management during pregnancy [46] and its ability to enable immediate adjustment to weight-related behaviours [47], initiating and maintaining this behaviour during preconception may lead to prevention of weight gain as well as significant improvements in weight management once a pregnancy does occur. Approximately 35% of women who consumed alcohol at baseline reported ceasing consumption altogether at evaluation, and a large proportion (approximately 80%) abstained from excessive drinking since starting the intervention. The prevalence of alcohol consumption and excessing drinking in women actively trying to conceive, without known participation in PCC or an intervention, has been reported at 85% and 56%, respectively [42]. The findings from other web-based preconception interventions indicate decreased alcohol consumption post intervention, highlighting strong motivation for behaviour change among women planning pregnancy and positive impacts of digital information provision without coaching support [48]. Women who excessively drink before pregnancy are at particular risk of drinking after becoming pregnant [49], and the preconception period is regarded a critical time to intervene, particularly for planned pregnancies [50]. Furthermore, alcohol intake is a risk factor for obesity in some individuals [51]. While our cohort reported lower alcohol consumption at baseline (57%) compared to previous studies in the literature [42], OptimalMe significantly decreased the number of women consuming alcohol as they approached pregnancy. Our findings align with other digital health interventions that have demonstrated effective preconception risk reduction [52]. Given the improvement in modifiable behaviours, digital interventions with health coaching may be an effective method to communicate risks and achieve behaviour change for women with the intention to conceive. These findings may extend to other areas of health promotion through digital interventions. However, due to the potential for pregnancy intentions to increase motivation, digital interventions need to be tested in different settings and populations.

OptimalMe provides a setting in which health coaches can inform and encourage behavioural and social change to optimise the health of women prior to pregnancy, and the online education modules can increase awareness of biomedical factors and encourage women into consultation with clinical care. It is promising that women demonstrate uptake of this low-intensity, non-prescriptive information provision. These results confirm that the provision of knowledge-enhancing tools and general healthy lifestyle information, combined with skilled health coaching focusing on small, sustainable improvements, can be an effective method for behaviour change and self-management. Critically, reaching a general population via a digital platform has the potential to improve equity and access for broader populations of women. Ninety-one percent of the Australian population are active internet users, and the internet is commonly used to obtain information [53]. Digital interventions present an opportunity to reach, promote and deliver PCC and lifestyle interventions to women thinking about or planning a pregnancy, who may not be engaged with health care. The suboptimal preconception health and behaviour of this cohort supports the need for enhanced efforts towards PCC on a population level. OptimalMe is fit for purpose to be used nationally as a whole-of-population approach to improving PCC. A further evaluation of engagement factors and scoping how to reach women outside of the private healthcare sectors are needed. However, OptimalMe demonstrates a feasible intervention for PCC.

## 6. Strengths and Limitations

Our rigorously developed questionnaire assessed an extensive range of health and lifestyle behaviours in accordance with the majority of national PCC recommendations [14]. Our stratification by health coaching delivery method strengthens the understanding of the impact of remotely delivered health and lifestyle interventions. While the self-reported nature of our data may be considered a limitation, OptimalMe is an adaptation of interventions with proven clinical outcomes [24]. Our approach prioritises testing feasibility for scalability over controlled clinical outcomes. This emphasis allows us to explore broader adoption and scalability. Utilising self-reported outcome methodologies becomes crucial for enhancing accessibility, expanding reach and increasing engagement within implementation science.

This program presents a promising opportunity for targeted, individual-level PCC, by exploring innovative channels, as recommended in the WHO action for PCC [54]. Our cohort consisted of women who had private health insurance, which may limit the generalisability of our results to other populations, owing to an overall higher socio-demographic profile. However, we studied a group of women from the general population who compare with Australian census data, and therefore our findings likely apply to most Australian women. Our evaluation had a response rate of 73%, which is potentially indicative of the remotely delivered design. This may have influenced our results but is unlikely to have led to bias [55]. The program is designed to be socially and culturally inclusive and to suit different levels of health literacy. However, the recruitment methods from this intervention did not enable us to test its impact in different groups. Further work is required to evaluate how the OptimalMe program meets the needs of LGBTQIA+, low-literacy, CaLD and Indigenous persons.

## 7. Conclusions

OptimalMe demonstrates that a low-intensity, non-prescriptive preconception health and lifestyle intervention in otherwise healthy women improved knowledge, behaviour and engagement with primary care. These improvements in lifestyle and adherence to PCC recommendations will have beneficial effects on the health of women and their children in the short and longer term. The findings of this study have important implications for equitable access to an evidence-based intervention for women in the preconception life phase.

## Figures and Tables

**Table 1:** Demographic characteristics (baseline).

		Health Coaching Group		
Characteristic	All	Phone	Video	*p*-Value
**Age (years) mean (SD)**	***n* = 298**	***n* = 153**	***n* = 145**	
	31.8 (4.3)	32.2 (4.4)	31.4 (4.2)	0.123
**Country of birth**	***n* = 298**	***n* = 153**	***n* = 145**	
Australia	211 (70.8)	102 (66.7)	109 (75.2)	0.106
Outside Australia	87 (29.2)	51 (33.3)	36 (24.8)	
**Ethnicity (identify as)**	***n* = 298**	***n* = 153**	***n* = 145**	
Asian *	54 (18.1)	34 (22.2)	20 (13.8)	0.093
European	80 (26.8)	40 (26.1)	40 (27.6)	
Indigenous Australian	3 (1.0)	3 (2.0)	0 (0.0)	
Oceanian **	119 (39.9)	59 (38.6)	60 (41.4)	
Other	42 (14.1)	17 (11.1)	25 (17.2)	
**Education**	***n* = 298**	***n* = 153**	***n* = 145**	
Bachelor degree & above	240 (80.5)	127 (83.0)	113 (77.9)	0.483
Certificate	19 (6.4)	10 (6.5)	9 (6.2)	
Diploma	24 (8.1)	10 (6.5)	14 (9.7)	
Year 10 or below	1 (0.3)	1 (0.7)	0 (0.0)	
Year 12 or equivalent	16 (4.7)	5 (3.3)	9 (6.2)	
**Working status**	***n* = 298**	***n* = 153**	***n* = 145**	
Casual/temporary work	12 (4.0)	6 (3.9)	6 (4.1)	0.637
Full time paid work	230 (77.2)	115 (75.2)	115 (79.3)	
No paid work	20 (6.7)	13 (8.5)	7 (4.8)	
Part time paid work	36 (12.1)	19 (12.4)	17 (11.7)	
**Weekly gross household income (AUD)**	***n* = 298**	***n* = 153**	***n* = 145**	
Less than AUD 999 per week (AUD 51,999 or less per year)	9 (3.0)	4 (2.6)	5 (3.4)	0.208
AUD 1000–1499 per week (AUD 52,000–77,999 per year)	28 (9.4)	13 (8.5)	15 (10.3)	
AUD 1500–1999 per week (AUD 78,000–103,999 per year)	32 (10.7)	21 (13.7)	11 (7.6)	
AUD 2000–2999 per week (AUD 104,000-155,999 per year)	70 (23.5)	32 (20.9)	38 (26.2)	
AUD 3000 or more per week (AUD 156,000 or more per year)	115 (38.6)	55 (35.9)	60 (41.4)	
I prefer not to answer	44 (14.8)	28 (18.3)	16 (11.0)	
**Marital status**	***n* = 298**	***n* = 153**	***n* = 145**	
Married or de facto	276 (92.6)	140 (91.5)	136 (93.8)	0.722
Never married or single	19 (6.4)	11 (7.2)	8 (5.5)	
Separated or divorced	3 (1.0)	2 (1.3)	1 (0.7)	
**Number of children**	***n* = 298**	***n* = 153**	***n* = 145**	
None (0)	257 (86.2)	128 (83.7)	129 (89.0)	0.213
One (1)	32 (10.7)	18 (11.8)	14 (9.7)	
Two (2)	5 (1.7)	3 (2.0)	2 (1.3)	
Three or more (≥3)	4 (1.3)	4 (2.6)	0 (0.0)	

* North-East Asian, South-East Asian, Southern and Central Asian. ** Non-Indigenous Australian Peoples, New Zealand Peoples, Polynesia, Micronesia, Melanesian and Papuan.

**Table 2:** Baseline preconception health conditions and behaviours.

		Health Coaching Group		
Characteristic/Factor or Action	All	Phone	Video	*p*-Value
**Weight (kg) mean (SD)**	***n* = 2989**	***n* = 153**	***n* = 145**	
	70.5 (17.7)	70.5 (18.4)	70.4 (16.9)	0.950
**BMI (kg/m^2^) mean (SD)**	***n* = 298**	***n* = 153**	***n* = 145**	
	25.7 (6.1)	25.9 (6.3)	25.7 (5.9)	0.644
**BMI category**	***n* = 298**	***n* = 153**	***n* = 145**	
Underweight	9 (3.0)	2 (1.3)	7 (4.8)	0.150
Healthy	163 (54.7)	91 (59.5)	72 (49.7)	
Overweight	68 (22.8)	31 (20.3)	37 (25.5)	
Obese	58 (19.5)	29 (19.0)	29 (20.0)	
**Weighing behaviour**	***n* = 298**	***n* = 153**	***n* = 145**	
Frequent	187 (62.8)	103 (67.3)	84 (57.9)	0.094
Infrequent	111 (37.2)	50 (32.7)	61 (42.1)	
**Chronic conditions/medical history**	***n* = 298**	***n* = 153**	***n* = 145**	
Asthma	40 (13.4)	19 (12.4)	21 (14.5)	0.601
Depression	38 (12.8)	17 (11.1)	21 (14.5)	0.383
Anxiety	66 (22.1)	31 (20.3)	35 (24.1)	0.421
Polycystic ovary syndrome (PCOS)	37 (12.4)	24 (15.7)	13 (9.0)	0.079
None	154 (51.7)	75 (49.0)	79 (54.5)	0.346
**Reproductive history**	***n* = 298**	***n* = 153**	***n* = 145**	
Diabetes in pregnancy (GDM)	5 (1.7)	3 (2.0)	2 (1.4)	0.696
Pre-eclampsia	3 (1.0)	3 (2.0)	0 (0.0)	0.090
Miscarriage/stillbirth	31 (10.4)	15 (9.8)	16 (11.0)	0.728
Birth defect(s)	3 (1.0)	2 (1.3)	1 (0.7)	0.594
Pre-term birth	6 (2.0)	5 (3.3)	1 (0.7)	0.113
**Genetic conditions (personal or family history)**	***n* = 297**	***n* = 153**	***n* = 144**	
No	162 (54.5)	87 (56.9)	75 (52.1)	0.602
Unsure	86 (29.0)	43 (28.1)	43 (29.9)	
Yes	49 (16.5)	23 (15.0)	26 (18.1)	
**Diagnosed iron/vitamin D nutrient deficiency (current or previous)**	***n* = 269**	***n* = 139**	***n* = 130**	
Iron	148 (55.0)	70 (50.4)	78 (60.0)	0.267
Vitamin D	112 (41.6)	56 (40.3)	56 (43.1)	0.845
Unsure	72 (26.8)	40 (28.8)	32 (24.6)	0.700
**Vaccines (up-to-date)**	***n* = 271**	***n* = 140**	***n* = 131**	
Measles, Mumps, Rubella (MMR)	239 (88.2)	124 (88.6)	115 (87.8)	0.923
Hepatitis B	230 (84.9)	123 (87.9)	107 (81.7)	0.344
Tetanus/Diphtheria/Pertussis (whooping cough)	221 (81.5)	119 (85.0)	102 (77.9)	0.299
**Immunisation status (in most recent flu season)**	***n* = 297**	***n* = 153**	***n* = 144**	
Influenza vaccine	184 (62.0)	95 (62.1)	89 (61.8)	0.588
**Immunisation status (virus/vaccine)**	***n* = 297**	***n* = 153**	***n* = 144**	
Chicken pox (Varicella)	276 (92.9)	141 (92.2)	135 (93.8)	0.510
**Cervical screening**	***n* = 297**	***n* = 153**	***n* = 144**	
Up-to-date	250 (84.2)	129 (84.3)	121 (84.0)	0.588
**Smoking status**	***n* = 298**	***n* = 153**	***n* = 145**	
No	284 (95.3)	145 (94.7)	139 (95.9)	0.402
No, I have stopped to prepare for pregnancy	9 (3.0)	4 (2.6)	5 (3.4)	
Yes	5 (1.7)	4 (2.6)	1 (0.7)	
**Alcohol**	***n* = 297**	***n* = 153**	***n* = 144**	
No	80 (26.9)	42 (27.5)	38 (26.3)	0.411
No, I have stopped to prepare for pregnancy	47 (15.8)	20 (13.1)	27 (18.8)	
Yes	170 (57.2)	91 (59.4)	79 (54.9)	
Recreational drug * use	***n* = 297**	***n* = 153**	***n* = 144**	
No	292 (98.3)	151 (98.7)	141 (97.9)	0.372
No, I have stopped to prepare for pregnancy	4 (1.3)	1 (0.7)	3 (2.1)	
Yes	1 (0.3)	1 (0.7)	0 (0.0)	
**Taking preconception supplement**	***n* = 297**	***n* = 153**	***n* = 144**	
Both folic acid and iodine	103 (34.7)	58 (37.9)	45 (31.3)	0.348
Folic acid (folate)	77 (25.9)	38 (24.8)	39 (27.1)	
Iodine	2 (0.7)	0 (0.0)	2 (1.4)	
None of the above	115 (38.7)	57 (37.3)	58 (40.3)	
**Using contraception**	***n* = 297**	***n* = 153**	***n* = 144**	
Yes	97 (32.7)	44 (28.8)	53 (36.8)	0.198

* (Cocaine/crack, marijuana, methamphetamines, methadone, heroin and ecstasy).

**Table 3:** Preconception health and lifestyle behaviours since OptimalMe intervention (evaluation).

		Health Coaching Group		
Factor or Action	All	Phone	Video	*p*-Value
**Weight (kg) mean (SD)**	***n* = 203**	***n* = 110**	***n* = 93**	
	69.8 (18.4)	70.5 (18.6)	68.9 (18.2)	0.531
**BMI (kg/m^2^) mean (SD)**	***n* = 203**	***n* = 110**	***n* = 93**	
	25.5 (6.3)	25.9 (6.2)	25.1 (6.3)	0.318
**BMI category**	***n* = 203**	***n* = 110**	***n* = 93**	
Underweight	9 (4.4)	1 (0.9)	8 (8.6)	0.031 *
Healthy	116 (57.1)	67 (60.9)	49 (52.7)	
Overweight	40 (19.7)	19 (17.3)	21 (22.6)	
Obese	38 (18.7)	23 (20.9)	15 (16.1)	
**Weighing behaviour**	***n* = 214**	***n* = 113**	***n* = 101**	
Frequent	180 (84.1)	94 (83.2)	86 (85.1)	0.670
Infrequent	34 (15.9)	19 (16.8)	15 (14.9)	
**Genetic testing**	***n* = 217**	***n* = 115**	***n* = 102**	
	42 (19.4)	28 (24.3)	14 (13.7)	0.0.92
**Smoking**	***n* = 217**	***n* = 115**	***n* = 102**	
	4 (1.8)	3 (2.6)	1 (1.0)	0.463
**Alcohol**	***n* = 217**	***n* = 115**	***n* = 102**	
Any consumption	99 (45.6)	56 (48.7)	43 (42.2)	0.406
Four (4) or more drinks in one sitting	27 (12.4)	16 (13.9)	11 (10.8)	0.516
**Taken recreational drugs**	***n* = 217**	***n* = 115**	***n* = 102**	
	1 (0.5)	0 (0.0)	1 (1.0)	0.367
Had any vaccine ** (excluding COVID)	***n* = 217**	***n* = 115**	***n* = 102**	
	73 (33.6)	35 (30.4)	38 (37.3)	0.369
**Cervical screening**	***n* = 217**	***n* = 115**	***n* = 102**	
	57 (26.3)	31 (27.0)	26 (25.5)	0.627
**STI screening**	**n = 217**	**n = 115**	**n = 102**	
	48 (22.1)	27 (23.5)	21 (20.6)	0.567
**Taken a preconception supplement**	***n* = 217**	***n* = 115**	***n* = 102**	
	158 (72.8)	86 (74.8)	72 (70.6)	0.509
**Taken a Vitamin D supplement**	***n* = 217**	***n* = 115**	***n* = 102**	
	107 (49.3)	64 (55.7)	43 (42.2)	0.091
**Visited GP for PCC**	***n* = 217**	***n* = 115**	***n* = 102**	
	158 (72.8)	79 (68.7)	79 (77.5)	0.228
**Improved lifestyle behaviours (any)**	***n* = 216**	***n* = 115**	***n* = 101**	
I did not need to	11 (5.1)	7 (6.1)	4 (4.0)	0.649
No	14 (6.5)	7 (6.1)	7 (6.9)	
Unsure	4 (1.9)	3 (2.6)	1 (1.0)	
Yes	187 (86.6)	98 (85.2)	89 (88.1)	
**Increased knowledge**	***n* = 216**	***n* = 115**	***n* = 101**	
Healthy food choices	108 (50.0)	58 (50.4)	50 (49.5)	0.820
Unhealthy food choices	88 (40.7)	49 (42.6)	39 (38.6)	0.476
Methods for physical activity	104 (48.1)	58 (50.4)	46 (45.5)	0.605

* No statistical significance after post hoc Bonferroni correction (*p* < 0.00625). ** Vaccine [EXCLUDING COVID]: (measles, mumps, rubella (MMR); hepatitis B; tetanus/diphtheria/pertussis (whooping cough); chicken pox; influenza (flu)).

## Data Availability

Data is available on request from the corresponding author (C.L.H.). The data are not publicly available due to privacy.
